# Effects of the Administration of 25(OH) Vitamin D3 in an Experimental Model of Chronic Kidney Disease in Animals Null for 1-Alpha-Hydroxylase

**DOI:** 10.1371/journal.pone.0170654

**Published:** 2017-01-20

**Authors:** Noelia Torremadé, Milica Bozic, David Goltzman, Elvira Fernandez, José M. Valdivielso

**Affiliations:** 1 Vascular and Renal Translational Research Group, REDinREN del ISCIII, IRBLleida, University Hospital Arnau de Vilanova, Lleida, Spain; 2 Calcium Research Laboratory, McGill University Health Center and Department of Medicine, McGill University, Montréal, Québec, Canada; University Medical Center Utrecht, NETHERLANDS

## Abstract

The final step in vitamin D activation is catalyzed by 1-alpha-hydroxylase (CYP27B1). Chronic kidney disease (CKD) is characterized by low levels of both 25(OH)D_3_ and 1,25(OH)_2_D_3_ provoking secondary hyperparathyroidism (2HPT). Therefore, treatments with active or native vitamin D compounds are common in CKD to restore 25(OH)D_3_ levels and also to decrease PTH. This study evaluates the dose of 25(OH)D_3_ that restores parathyroid hormone (PTH) and calcium levels in a model of CKD in CYP27B1^-/-^ mice. Furthermore, we compare the safety and efficacy of the same dose in CYP27B1^+/+^ animals. The dose needed to decrease PTH levels in CYP27B1^-/-^ mice with CKD was 50 ng/g. That dose restored blood calcium levels without modifying phosphate levels, and increased the expression of genes responsible for calcium absorption (TRPV5 and calbindinD- 28K in the kidney, TRPV6 and calbindinD-9k in the intestine). The same dose of 25(OH)D_3_ did not modify PTH in CYP27B1^+/+^ animals with CKD. Blood calcium remained normal, while phosphate increased significantly. Blood levels of 25(OH)D_3_ in CYP27B1^-/-^ mice were extremely high compared to those in CYP27B1^+/+^ animals. CYP27B1^+/+^ animals with CKD showed increases in TRPV5, TRPV6, calbindinD-28K and calbindinD-9K, which were not further elevated with the treatment. Furthermore, CYP27B1^+/+^ animals displayed an increase in vascular calcification. We conclude that the dose of 25(OH)D_3_ effective in decreasing PTH levels in CYP27B1^-/-^ mice with CKD, has a potentially toxic effect in CYP27B1^+/+^ animals with CKD.

## Introduction

Vitamin D is a major regulator of Ca^2+^ and phosphate homeostasis and it is essential for proper development and maintenance of bones.[1] The active form of vitamin D, 1,25(OH)_2_D_3_, is synthesized from its precursor 25OHD_3_ by the 25-hydroxyvitamin-D-1α-hydroxylase (1-α-hydroxylase; CYP27B1).[2] Mutations in the CYP27B1 gene cause severe disorders of Ca^2+^ homeostasis, including vitamin D-dependent rickets type I (VDDRI).[3]

Chronic kidney disease- mineral and bone disorder (CKD-MBD) is a common problem in patients with kidney disease. It is characterized by abnormal levels of calcium (Ca) and phosphate (P) and biochemical alterations of mineral metabolism related hormones, alongside vascular calcification. Among those biochemical alterations, low levels of active vitamin D metabolites are of paramount importance.[4] To treat CKD-MBD related complications, vitamin D compounds have been widely used. However, the use of active vitamin D compounds such as 1,25(OH)_2_D_3_ has been accompanied by undesired side effects like hypercalcemia and hyperphosphatemia, which increase the risk of vascular calcification.[5] To avoid these effects, vitamin D analogs were developed in order to suppress PTH secretion with a minimal calcemic action. [6–8]

Active vitamin D compounds directly increase intestinal and renal Ca^2+^ reabsorption through up-regulation of Ca^2+^ transport proteins.[9] A distinct family of epithelial Ca^2+^ channels (TRPV6 and TRPV5) has been identified, which provides the molecular identity of the apical entry mechanism facilitating this active Ca^2+^ reabsorption process.[10, 11] Ca^2+^ entry via these Ca^2+^ channels is followed by cytosolic diffusion facilitated by Ca^2+^ binding proteins (calbindin-D28K and /or calbindin-D9k) and active extrusion of Ca^2+^ across the basolateral membrane by a high affinity plasma membrane Ca^2+^-ATPase (PMCA1b) and Na^+^-Ca^2+^ exchanger (NCX1). In this active process, TRPV5 and TRPV6 probably form the final target for hormonal control, suggesting that these channels could be the primary targets in the regulation of Ca^2+^ reabsorption.[9]

Not only 1,25(OH)_2_D_3_, but also 25OHD_3_ levels are low in CKD-MBD. Due to the existence of CYP27B1 in many tissues, international guidelines propose the correction of low 25OHD_3_ levels to maintain the pleiotropic beneficial effects of vitamin D.[12, 13] Thus, the use of cholecalciferol or even 25OHD_3_ to correct its own deficiency has been recommended.[14] Furthermore, the normalization of 25OHD_3_ levels has been reported to have an effect in decreasing PTH levels.[15] It has been also shown that 25OHD_3_ can mediate its effects independent of its conversion to 1,25(OH)_2_D_3_.[16] However, it is unknown whether the effect of 25OHD_3_ on PTH reduction is achieved directly by activation of VDR or by residual conversion into 1,25(OH)_2_D_3_.

The hypothesis of the present study is that the dose necessary to decrease PTH with 25OHD_3_ (without conversion into 1,25(OH)_2_D_3_) in CKD is very high, and could have toxic effects. Thus, the aim of the present study was to find the dose of 25OHD_3_ effective enough to restore Ca^2+^ and PTH levels in a CYP27B1 knockout (CYP27B1^-/-^) mouse model with CKD, therefore independently of conversion into 1,25(OH)_2_D_3_, and to test the same dose in CYP27B1^+/+^ mice to evaluate its safety and efficacy in decreasing PTH. We also investigated the effects of 25OHD_3_ treatment in regulating the expression of the main genes responsible for intracellular and paracellular calcium transport in the kidney and intestine of the same mice.

## Methods

### *In Vivo* study

All animal studies were approved by the University of Lleida Animal Ethics Committee in accordance with the guidelines of European Research Council for the care and use of laboratory animals. In all surgical procedures performed in animals, isoflurane was used as anesthetic and buprenorphine was used as analgesic after the surgery.

### Generation and characterization of CYP27B1^-/-^ mice

CYP27B1^-/-^ mice were provided by Dr. David Goltzman (Montreal, Canada) and were generated by ablation of exon 6 to exon 9 (26). These mice were bred to C57/BL6 wild-type animals and the heterozygous offspring were crossed to produce CYP27B1^-/-^ (KO) and CYP27B1^+/+^ (WT) animals. KO mice were fed a rescue diet (2% calcium, 1.25% phosphorus, 20% lactose and 2.2 IU/g vitamin D3; Harlan Teklad, TD.96348) during growth and maintenance. Before starting the experimental process the diet was changed to a standard mouse chow for KO animals (0.6% Ca^2+^, 0.8% phosphorus, and 0.6 IU/g vitaminD_3_; Harlan Teklad), while WT animals were maintained on a 0.9% phosphorus diet in order to induce an increase in PTH levels.

### Model of CKD in mice

Subtotal nephrectomy was performed in 10 week-old mice after the two-step surgical procedure for 75% nephron reduction (NX), as previously described. [17] Briefly, the parenchyma of the left kidney was reduced 50%. The kidney was exposed, decapsulated and carefully cauterized, reducing the parenchyma of the upper and the lower pole. After 1-week of recovery period, right-sided total nephrectomy was performed. Treatments started two weeks after nephrectomy to facilitate recovery after the operation.

First, a dose response analysis was carried out in 10 week–old nephrectomized CYP27B1^-/-^ mice (KO NX) using 25, 50, 100 ng/g of 25OHD_3_ (Sigma-Aldrich) administered intraperitoneally three times per week for 30 days (n = 8 per dose). Terminal blood samples were taken 24 hours after the last injection. In parallel, an additional group of KO NX mice (n = 8) was treated with a dose of 1,25(OH)_2_D_3_ (50 pg/g) three times per week for 30 days and a terminal blood sample was taken 24 hours after the last injection. The goal was to determine the dosage of 25OHD_3_ that induced changes in PTH levels similar to those achieved with 1,25(OH)_2_D_3_. After selecting the dosage of 50 ng/g for 25OHD_3_, nephrectomized CYP27B1^+/+^ mice (WT NX, n = 8) were treated with the same dose and route of administration for a 30 days period. A group of sham-operated mice were used as a control in the present study (CYP27B1^+/+^ (WT control) n = 6, CYP27B1^-/-^ (KO control) n = 5).

Euthanasia was performed 24 hours after the last injection. Then, a terminal blood sample was collected and aortas were divided in two parts, one frozen in liquid nitrogen for calcium content determination and the other one fixed in formalin solution followed by processing and paraffin embedding. Kidney and duodenum were collected to study the expression of calcium transport proteins.

### Serum biochemical analysis

Blood was collected by cardiac puncture and centrifuged at 2500 rpm for 10 min at 4°C to obtain serum.

Ca^2 +^ and P were analyzed by a standard colorimetric assay analysis in the Biochemistry service of the Arnau de Vilanova Hospital (HUAV) in Lleida using a multichannel autoanalyzer (Roche/Hitachi Modular Analytics), using the following methods: 1) for calcium the o-cresolphthalein complexone method, 2) for phosphate the ammonium molybdate method. Blood urea nitrogen (BUN) was determined by colorimetric assay using the QuantiChrom Urea assay kit (DIUR-500, Gentaur, San Jose, CA, USA). Immunoassays were used to determine 25OHD_3_, 1,25 (OH)_2_D_3_ (IDS 25OHD_3_ EIA, Immunodiagnostic Systems, The Boldons, UK), and also PTH (PTH mouse ELISA kit, Immunotopics, San Clemente, CA, USA).

### Quantitative analysis of aortic calcium

Aortic tissue was desiccated for 20–24 hours at 60°C, crushed to a powder with a pestle and mortar, and decalcified with HCl (1N) at 4°C, and then vortexed for 16 hours. After centrifugation, supernatant was collected and calcium content determined colorimetrically using the o-cresolphthalein complexone method, whereas total protein content was determined by the Lowry method (Bio-Rad, Hercules, CA, USA), as previously described.[7] Aortic calcium content was normalized by the protein amount in the sample and expressed as ng of Ca/ mg of protein.

### Histology and immunohistochemistry

Immunostaining for CalbindinD28k and TRPV5 were carried out on 5-μm-thick kidney tissue sections. Sections were deparaffinized through xylene and rehydrated through graded ethanol concentrations into distilled water, as previously described.[18] Shortly, antigen retrieval was done by boiling the slides in 10mM citrate buffer (pH 6) for 10 minutes. Endogenous peroxidase quenching (30 min incubation in 0.66% (vol/vol) H_2_O_2_/PBS) was followed by blocking of nonspecific binding with normal horse blocking serum (Vector Laboratories) for 30 min at room temperature (RT). Anti-rabbit calbindinD28k (1:500) and anti-guinea pig TRPV5 antibody (1:1500) were incubated overnight at 4°C. After washing with PBS, slides were treated with goat anti-rabbit Alexa 488 (1:300) (for CalbindinD28k) and Cy2 AffiniPure donkey anti-guinea pig IgG (1:150) (for TRPV5) for 1 hour at RT. Sections were dehydrated in methanol and mounted with Mowiol. Negative controls were performed by incubation with nonimmune serum in place of a specific antibody, which resulted in complete absence of staining. Images were taken with Zeiss fluorescence microscope with a digital camera (Nikon DMX1200).

For calcium staining in aortic sections, samples were deparaffinized, rehydrated and stained in 2% Alizarin red solution (Sigma A3757, Sigma Aldrich, SL, MO, USA) at pH between 4,1–4,3 for 5 min. After staining, samples were rehydrated with acetone, acetone-xylene (1:1), xylene and mounted in synthetic mounting medium (DPX, Sigma Aldrich, SL, MO, USA).

### Real time PCR

Total RNA was extracted from the kidney and duodenum samples using TRIzol reagent (Sigma Aldrich, SL, MO, USA) and following manufacturer’s instructions. The RNA concentrations were determined by nanodrop (ND-100) spectrophotometer. Reverse transcription was performed as previously described.[19] Real time PCR with gene-specific SYBR Green primers for mouse TRPV5 (5’CTGGAGCTTGTGGTTTCCTC3’), Calbindin-D28k (5’GACGGAAGTGGTTACCTGGA3’), Calbindin-D9k (5’CCTGCAGAAATGAAGAGCATTTT3’), PMCA1b (5’GTCACCGGCCTTACGTGTAT3’), NCX1 (5’GTGACTGCCGTTGTGTTTGT3’), TRPV6 (5’GGCCTCACAACCTCATTTAC3’) and GAPDH (5’TAACATCAAATGGGGTGAGG3’) was performed with a CFX Real-Time PCR detection system (Bio-Rad Laboratories, S.A., Madrid, Spain). Forty cycles at 95°C for 15 seconds and 60°C for 1 minute were performed. Duplicate readings were taken, and the average was calculated. The relative mRNA levels were calculated by standard formulae (ΔΔCt method) using mouse GAPDH as an endogenous control. The results referred to a randomly selected basal sample that was considered as value = 1.

### Western blot analysis

Kidney tissue was homogenized in lysis buffer (50 mM HEPES, 250 mM NaCl, 5 mM EDTA, 0,1% Nonidet P-40) using polytron homogenizer. After centrifugation for 10 min at 10000 rpm, 4°C, supernatant was collected and protein concentration was determined using a BCA protein assay kit (Bio-Rad). 25μg of proteins were electrophoresed on 12% SDS-PAGE gels and transferred to PVDF membranes (Immobilon-P, Millipore), as previously described.[20] Membranes were probed with primary antibodies against CalbindinD28k (1:10000) and α-tubulin (1:5000) over night at 4°C. Appropriate horseradish peroxidase-conjugated secondary antibodies were used at 1:10000. The immunoreaction was visualized using chemiluminescent kits EZ ECL (Biological Industries) or ECL Advanced (Amersham Biosciences). Images were digitally acquired by Chemidoc (Bio Rad). Positive immunoreactive bands were quantified by densitometry and compared with the expression of adequate loading control (α-tubulin).

### Statistical analysis

Differences between groups were assessed by one-way ANOVA and Bonferroni posthoc test. Differences between WT and KO mice were assessed by two-way ANOVA and Bonferroni posthoc test. A p<0.05 was considered statistically significant. All data examined are expressed as mean ± SEM.

## Results

### 25OHD_3_ restores calcium and PTH levels in CYP27B1^-/-^ mice with CKD

**Fig 1:**
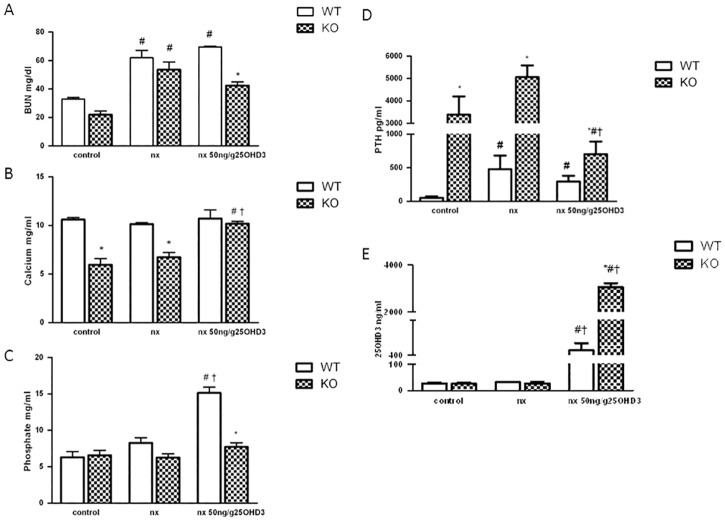
25OHD_3_ restores calcium and PTH levels in CYP27B1^-/-^ mice with CKD. **A**: BUN **B**: Calcium **C**: Phosphate **D**: PTH and E: 25OHD_3_ serum levels from CYP27B1^+/+^ (WT) and CYP27B1^-/-^ (KO) control mice, nephrectomized (nx) and nephrectomized treated with 50 ng/g of 25(OH)D_3_ for 1 month. Data are mean ± SEM * p< 0,001 vs. WT, # p< 0,05 vs. Control, † p< 0,05 vs. Nx, (n = 5 to 7).

We first sought to determine the dose of 25OHD_3_ effective enough to maintain serum Ca^2+^ and PTH levels in CYP27B1^-/-^ mouse model with CKD. CYP27B1^-/-^ (KO) mice were subjected to subtotal nephrectomy (KO NX) and treated with 25, 50 and 100 ng/g of 25OHD_3_ three times per week for 30 days. The dose of 25OHD_3_ that was able to normalize serum Ca^2+^ and reduce PTH levels in KO NX animals similar to 1,25OH_2_D_3_ (50 pg/g) was 50 ng/g of 25OHD_3_ (S1 Fig). This dose was chosen for further investigation in CYP27B1^+/+^ (WT) and CYP27B1^-/-^ (KO) mice. After subtotal nephrectomy, serum BUN levels increased in all nephrectomized groups of mice (Fig 1A), suggesting a similar degree of renal impairment. Calcium levels (Fig 1B), generally lower in KO mice (both in normal and NX conditions), increased with 25OHD_3_ treatment reaching similar levels as in WT animals. Neither nephrectomy nor 25OHD_3_ treatment did modify serum calcium levels of WT animals (Fig 1B). Phosphate levels, which were comparable in control and NX groups of mice, significantly increased only in WT NX animals treated with 25OHD_3_ (Fig 1C). Levels of PTH, which are very high in KO mice, increased even more after the nephrectomy and were partially corrected in KO NX mice after the treatment with 25OHD_3_ (Fig 1D). 25OHD_3_ had no effect on elevated PTH levels in WT NX mice (Fig 1D). Serum levels of 25OHD_3_ increased in both groups of mice treated with 25OHD_3_, but were higher in KO than in WT mice (Fig 1E). Circulating 1,25OH_2_D_3_ levels slightly increased in WT animals treated with 50 ng/g of 25OHD_3_ (WT NX: 69.49 ± 23.9; WT NX+25OH: 81,64 ± 15 pg/ml. p:0.6).

### 25OHD_3_ does not induce vascular calcification in CYP27B1^-/-^ mice with CKD

**Fig 2:**
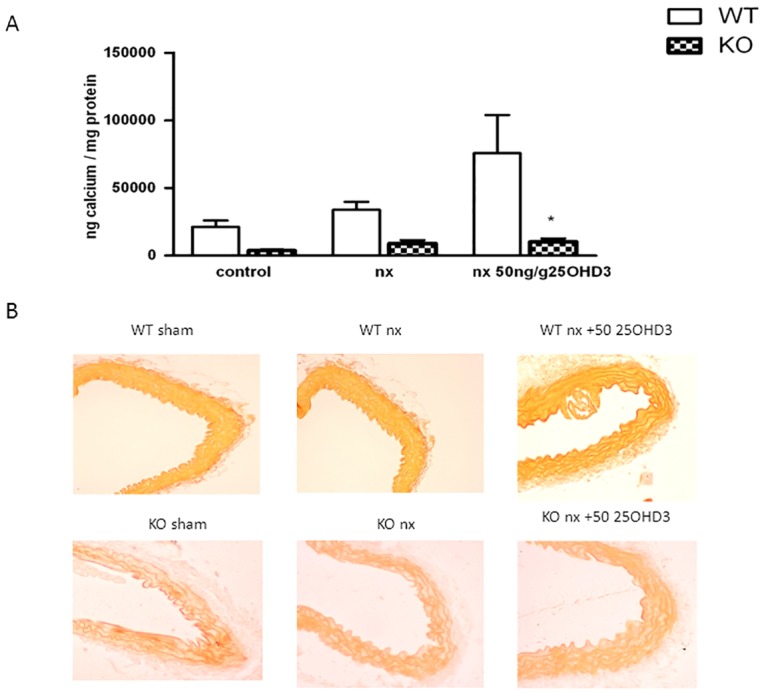
25OHD_3_ does not induce vascular calcification in CYP27B1^-/-^ mice with CKD. **A**: Calcium content in abdominal aorta normalized to protein in CYP27B1^+/+^ (WT) (white bars) and CYP27B1^-/-^ (KO) control mice, nephrectomized (nx) and nephrectomized treated with 25OHD3 (50ng/g) for 1 month. **B**: Representative histochemical of alizarin red staining from aortas of mice from the same groups. Data are mean ± SEM (n = 5 to 7) * p< 0,001 vs WT mice. † p< 0,05 vs. nx.

The effect of 25OHD_3_ on vascular calcification in WT and KO mice is shown in Fig 2. Treatment with 25OHD_3_ showed a tendency to increase calcium content in arteries of WT mice (Fig 2A), but did not modify calcium content in KO animals. Representative histochemical alizarin red staining from aortas showed an increase of the staining in the elastic laminas of WT mice treated with 25OHD_3_ (Fig 2B).

### 25OHD_3_ increases expression of TRPV6 and Calbindin-D9k in duodenum of CYP27B1^-/-^ mice with CKD

**Fig 3:**
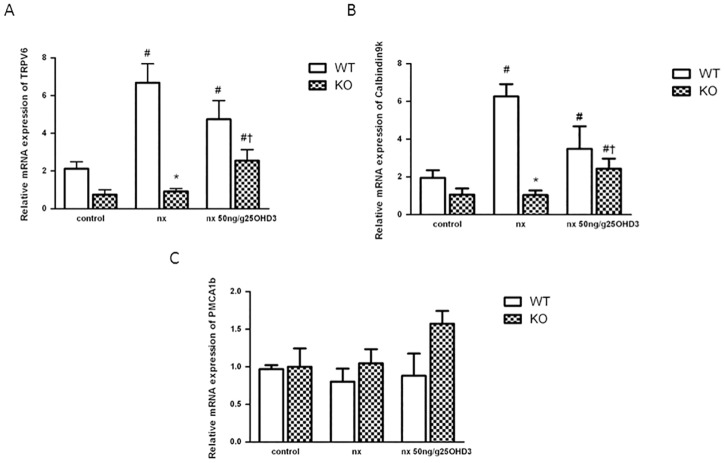
25OHD_3_ increases expression of TRPV6 and Calbindin-D9k in duodenum of CYP27B1^-/-^ mice with CKD. **A**: TRPV6 **B**: Calbindin-D9K and **C**: PMCA1b mRNA expression (qPCR) in CYP27B1^+/+^ (WT) and CYP27B1^-/-^ (KO) control mice, nephrectomized (nx) and nephrectomized treated with 25OHD3 for 1 month. Data are mean ± SEM * p< 0,001 vs. WT, # p< 0,05 vs. Control, † p< 0,05 vs. Nx, (n = 5 to 7).

We further investigated whether treatment with 25OHD_3_ had influence on the expression of genes encoding Ca^2+^ transport proteins involved in duodenal transcellular Ca^2+^ absorption. Administration of 50 ng/g of 25OHD_3_ produced a 3-fold increase in TRPV6 mRNA in KO NX mice, while in WT animals the increase of TRPV6 mRNA was already evident after the nephrectomy and did not further increase with the treatment (Fig 3A). Calbindin-D9k mRNA levels showed a similar profile (Fig 3B). The expression of PMCa1b mRNA was not significantly modified by any of the conditions (Fig 3C).

### 25OHD_3_ increases expression of Ca^2+^ transport genes and proteins in the kidney of CYP27B1^-/-^ with CKD

**Fig 4:**
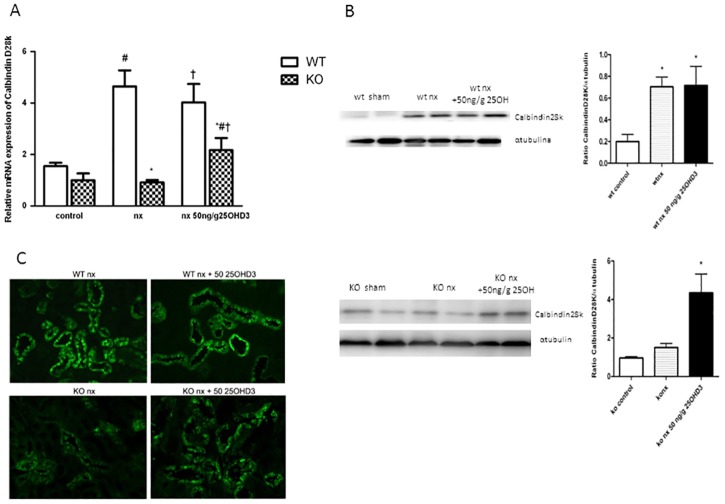
25OHD_3_ increases expression of CalbindinD-28K in the kidneys of CYP27B1^-/-^ mice with CKD. **A**: mRNA expression (qPCR) **B**: protein expression (western blot) and **C**: immunohistochemistry of CalbindinD-28K in CYP27B1^+/+^ (WT) and CYP27B1^-/-^ (KO) control mice, nephrectomized (nx) and nephrectomized treated with 25OHD3 for 1 month. Data are mean ± SEM * p< 0,001 vs. WT, # p< 0,05 vs. Control, † p< 0,05 vs. Nx, (n = 5 to 7).

The effect of 25OHD_3_ on mRNA expression of Ca^2+^ transport genes (TRPV5, Calbindin-D28k, NXC1 and PMCA1b) in the kidney was investigated by real-time quantitative PCR. The profile of Calbindin-D28k mRNA expression in the kidney was similar to the expression of Calbindin-D9k mRNA in the duodenum. Namely, nephrectomy (NX) increased Calbindin-D28k mRNA expression in the kidneys of WT mice, which was not further increased by 25OHD_3_ treatment (Fig 4A). On the contrary, nephron reduction in KO mice did not modify the expression of Calbindin-D28k mRNA, but the treatment with 25OHD_3_ managed to increase the expression of this gene in the kidney, although to the levels lower than in WT animals (Fig 4A). Analysis of protein expression, determined by Western blot (Fig 4B), corroborated results obtained by RT-PCR analysis. Furthermore, immunofluorescence analysis showed an increase of Calbindin-D28k staining in distal tubules of KO NX mice treated with 25OHD_3_ (Fig 4C).

**Fig 5:**
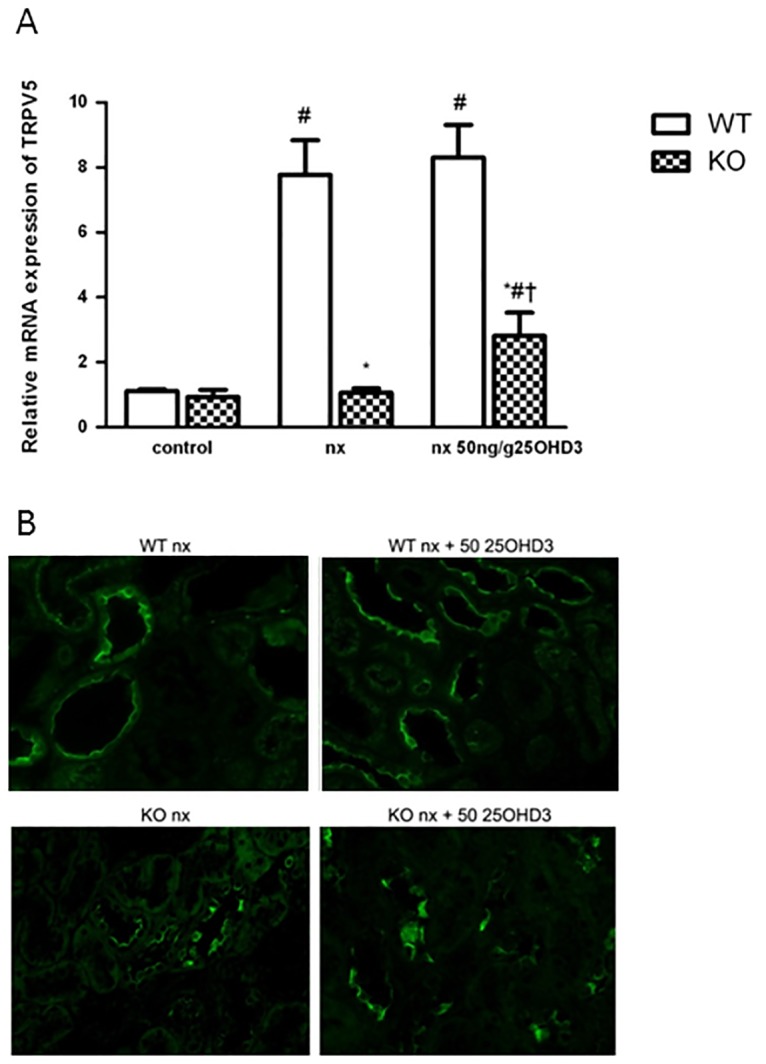
25OHD_3_ increases expression of TRPV5 in the kidneys of CYP27B1^-/-^ mice with CKD. **A**: mRNA expression (qPCR) and **B**: immunohistochemistry of TRPV5 in CYP27B1^+/+^ (WT) and CYP27B1^-/-^ (KO) control mice, nephrectomized (nx) and nephrectomized treated with 25OHD3 for 1 month. Data are mean ± SEM * p< 0,001 vs. WT, # p< 0,05 vs. Control, † p< 0,05 vs. Nx, (n = 5 to 7).

**Fig 6:**
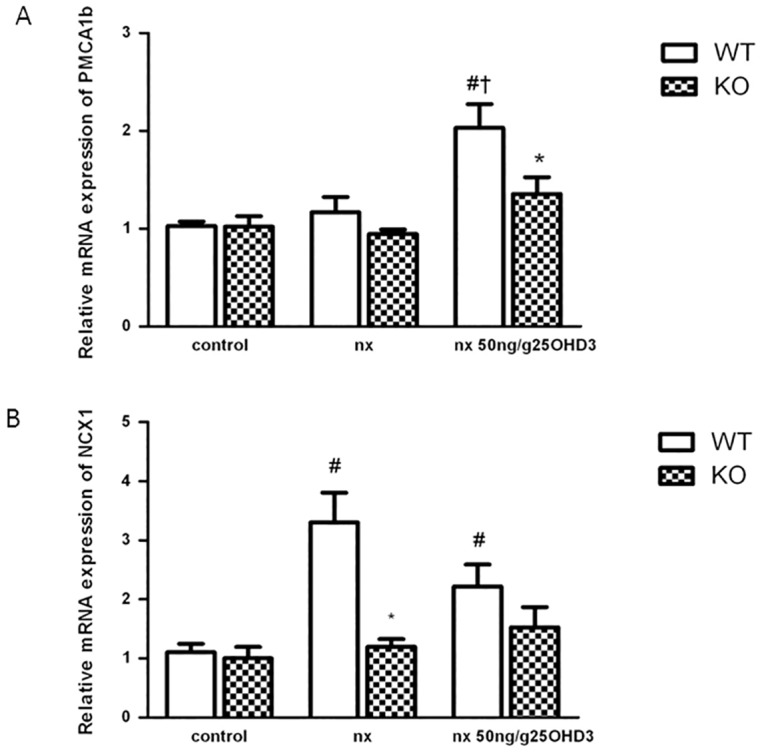
The effect of 25OHD3 on the expression of renal PMCA1b and NCX1 in CYP27B1^+/+^ and CYP27B1^-/-^ mice. **A**: mRNA expression (qPCR) of PMCA1b and **B**: NCX1 in CYP27B1^+/+^ (WT) and CYP27B1-/- (KO) control mice, nephrectomized (nx) and nephrectomized treated with 25OHD3 for 1 month. Data are mean ± SEM * p< 0,001 vs. WT, # p< 0,05 vs. Control, † p< 0,05 vs. Nx, (n = 5 to 7).

Expression of TRPV5 significantly increased in the WT group after the NX and did not further increase with the 25OHD_3_ treatment (Fig 5A). In the KO group, NX did not modify the expression of TRPV5 mRNA, but the treatment with 25OHD_3_ was able to increase the expression of this gene (Fig 5A). Immunofluorescence analysis showed an increase of TRPV5 staining in distal convoluted and connecting tubules of KO NX mice treated with 25OHD_3_ (Fig 5B). Expression of the basolateral extrusion genes (NCX1 and PMCA1b) was not affected by 25OHD_3_ (Fig 6A and 6B) in KO NX mice. Nevertheless, WT NX mice showed an increase of PMCA1b mRNA expression after administration of 25OHD_3_ treatment (Fig 6A), while the expression of NCX1 increased in the both WT NX groups (untreated and treated with 25OHD_3_) (Fig 6B).

## Discussion

In the present paper we demonstrate that the suppression of PTH in an experimental model of CKD can be achieved directly by 25OHD_3_ without its conversion to 1,25(OH)_2_D_3_, but the blood levels necessary to attain the effect are extremely high. Furthermore, although the effect is undoubtedly due to an effect of 25OHD3, it is unclear whether this effect is directly stimulating VDR in the parathyroid gland or indirectly by increasing calcium absorption in the intestine. Thus, the administration of 50 ng/g of 25OHD_3_ in KO NX animals decreased serum PTH to levels below the ones observed in the same animals with normal renal function. This inhibition was achieved with blood levels of 25OHD_3_ around 7 times higher than the normal levels and in parallel to a normalization of blood calcium levels. Thus, the decrease of PTH could be attained by a combination of normalization of blood calcium levels and direct activation of VDR by 25OHD_3_ in the parathyroid gland.

The results of the present study demonstrate that 25OHD_3_ can activate VDR in uremic conditions, and agree with previous results showing similar results in animals with normal renal function.[21] It has been shown that uremic milieu contains toxins that block the binding of activated VDR with VDRE in the promoter of target genes.[22] Thus, inhibition of VDR target gene expression by uremia can partially explain the resistance to 1,25(OH)_2_D_3_ observed in CKD. Our results show that reaching levels of 25OHD_3_ in blood high enough can overcome the mentioned problem. We demonstrate that treatment with 25OHD_3_ increased the expression of calcium channels in the kidney and intestine, most likely by direct effect of activated VDR on the promoter of target genes.

Furthermore, the administration of the same dose of 25OHD_3_ to WT NX animals showed interesting results. Namely, the dose administered was unable to significantly reduce the increase of PTH levels induced by the nephrectomy and high phosphate feeding. Although the PTH levels obtained with the nephrectomy in WT NX animals were far lower than measured in KO NX animals, there are similar to the ones found in CKD patients.[23] In any case, this different behavior of the treatment could be due to two reasons. On the one hand, the levels of 25OHD_3_ achieved in blood of WT NX mice were much lower than the ones observed in the KO NX animals. This effect could be explained by a higher rate of degradation of 25OHD_3_ or by its conversion to 1,25(OH)_2_D_3_. The degradation of both, 1,25(OH)_2_D_3_ and 25OHD_3_ is mediated by the enzyme 24-hydroxylase, the levels of which are highly induced by active vitamin D compounds.[24] Thus the levels of 24-hydroxylase are expected to be very low in the KO animals, increasing the half-life of the 25OHD_3_ and its blood concentration. Furthermore, and in contrast to KO mice, part of the 25OHD_3_ administered to the WT animals can be converted to 1,25(OH)_2_D_3_, which will also decrease 25OHD_3_ levels. Although this represents a very small portion of 25OHD_3_, the circulating levels of 1,25(OH)_2_D_3_ did not significantly increase, it could also have an influence on 25OHD_3_ blood levels. On the other hand, the administration of 25OHD_3_ to the WT animals induced an increase in the levels of phosphate which have been shown to stimulate PTH synthesis and release.[25] This effect can also be explained by the partial conversion of 25OHD_3_ into 1,25(OH)_2_D_3_ and subsequent activation of intestinal VDR, which has been shown to increase phosphate absorption from the intestine, although this effect should minor since circulating levels of 1,25(OH)_2_D_3_ did not significantly increase.[26]

Furthermore, the administration of the same doses of 25OHD_3_, although ineffective to decrease PTH levels, showed a tendency to increase vascular calcification. Thus, calcium levels in arteries of WT NX animals treated with 25OHD_3_ were significantly increased compared to the treated KO NX mice. The Alizarin red staining, although not showing an evident increase in vascular calcification, showed an increase in the red staining of the elastic laminas, suggesting an increase in calcium deposition in that area. The involvement of elastic lamina in medial calcification in patients, although challenged in the past, seems to be accepted nowadays.[27, 28] This pattern is also found in some genetically modified mice affected by extensive medial vascular calcification.[29, 30] The increase of vascular calcification in WT animals can be explained by the increase in P levels. It has been shown that high P levels are of paramount importance in the genesis of vascular calcification. Thus, increases in blood P have been shown to be associated with vascular calcification both in patients and in experimental animals.[31] Furthermore, a recent paper from our laboratory has shown that the upregulation of local CYP27B1 in the vascular smooth muscle cells is of paramount importance in the genesis of uremic vascular calcification.[17] Thus, in KO animals the absence of CYP27B1 blocks the increase in vascular calcification. However, in WT animals the administration of 25(OH)D_3_ could exacerbate the pro-calcificant effects of uremia by increasing the substrate for local production of 1,25(OH)_2_D_3_ in the artery.

We have also shown an effect of the uremia and the treatment with 25OHD_3_ on the expression of the proteins related to calcium transport both in the duodenum and in the kidney. The effect of the 25OHD_3_ treatment on the expressions of TRPV5, TRPV6, calbindins D9K and 28K in KO NX animals was similar. Thus NX did not have an effect of the levels of any of the proteins, but the treatment with 25OHD_3_ significantly increased its expression. Several VDRE have been identified in both TRPV and calbindin gene promoters.[32–35] Thus, the extremely high levels of 25OHD_3_ reached in the treated KO NX animals could be able to activate the VDRE in the promoter of the genes. In the WT animals however, the NX already increased the expression levels of all four genes. The effect of CKD on renal calcium transporter has been recently described.[36] The increase of proteins related to calcium transport has been attributed to a possible effect of PTH, FGF23 or even vitamin D. Our results show that active vitamin D must be playing a central role in this increase, because the effect is absent in KO animals. Furthermore, this is the first report showing that experimental CKD also induces an increase in duodenal calcium transport mechanisms. In the WT NX animals, the increase of expression of all four genes was not further induced by the administration of 25OHD3, either because the expression was already submaximal or because the levels of 25OHD_3_ achieved in the WT NX animals were insufficient to further increase the promoter activity of those four genes. The effects of uremia and the treatments on NCX1 followed a similar pattern. However, the expression of PMCA1b on both tissues was not affected in the same way, suggesting a different regulation of this gene. Thus, and in agreement with previous results, renal levels were increased by treatment with vitamin D.[37] However, intestinal levels of PMCA1b were not affected in any of the conditions confirming that, as it has been suggested previously, vitamin D effects on PMCA1b gene could be tissue specific.[38]

As any study in genetically modified animals, the main strength of the study relays in the fact that we can be absolutely sure that all the effects observed in the treatments are due to a direct effect of 25OHD_3_, as conversion to the active metabolite is totally blocked. The main limitation is that we did not collect parathyroid glands in the treated animals, and we can not differentiate whether the PTH reduction effect is mediated by direct effects on VDR in the parathyroid gland or in intestinal cells.

In conclusion, 25OHD_3_ is able to reduce PTH in a CYP27B1^-/-^ mouse model of CKD without direct conversion to active vitamin D, but the blood levels needed are extremely high. Although this effect on PTH is due to an increase of VDR signaling by 25OHD3, it could be a combination of a direct effect in the parathyroid gland and an induction of the calcium absorption machinery in the intestine, and the subsequent normalization of Ca blood levels.

## Supporting Information

S1 Fig Preliminary dose-response experiments. Levels of Ca (A), P (B) and PTH (C) in sham-operated KO animals, NX KO animals and NX KO animals treated with 50 pg/g of 1,25(OH)_2_D_3_ or 25, 50 and 100 ng/g of 25OHD_3_. *: p<0.01 vs KO sham. #: p<0.01 vs KO NX.
(TIF)
