# Supplementary figures and images for: Effects of the Administration of 25(OH) Vitamin D3 in an Experimental Model of Chronic Kidney Disease in Animals Null for 1-Alpha-Hydroxylase
Source: PLoS One. 2017 Jan 20;12(1):e0170654. doi: 10.1371/journal.pone.0170654 (PMC5249163; doi:10.1371/journal.pone.0170654)

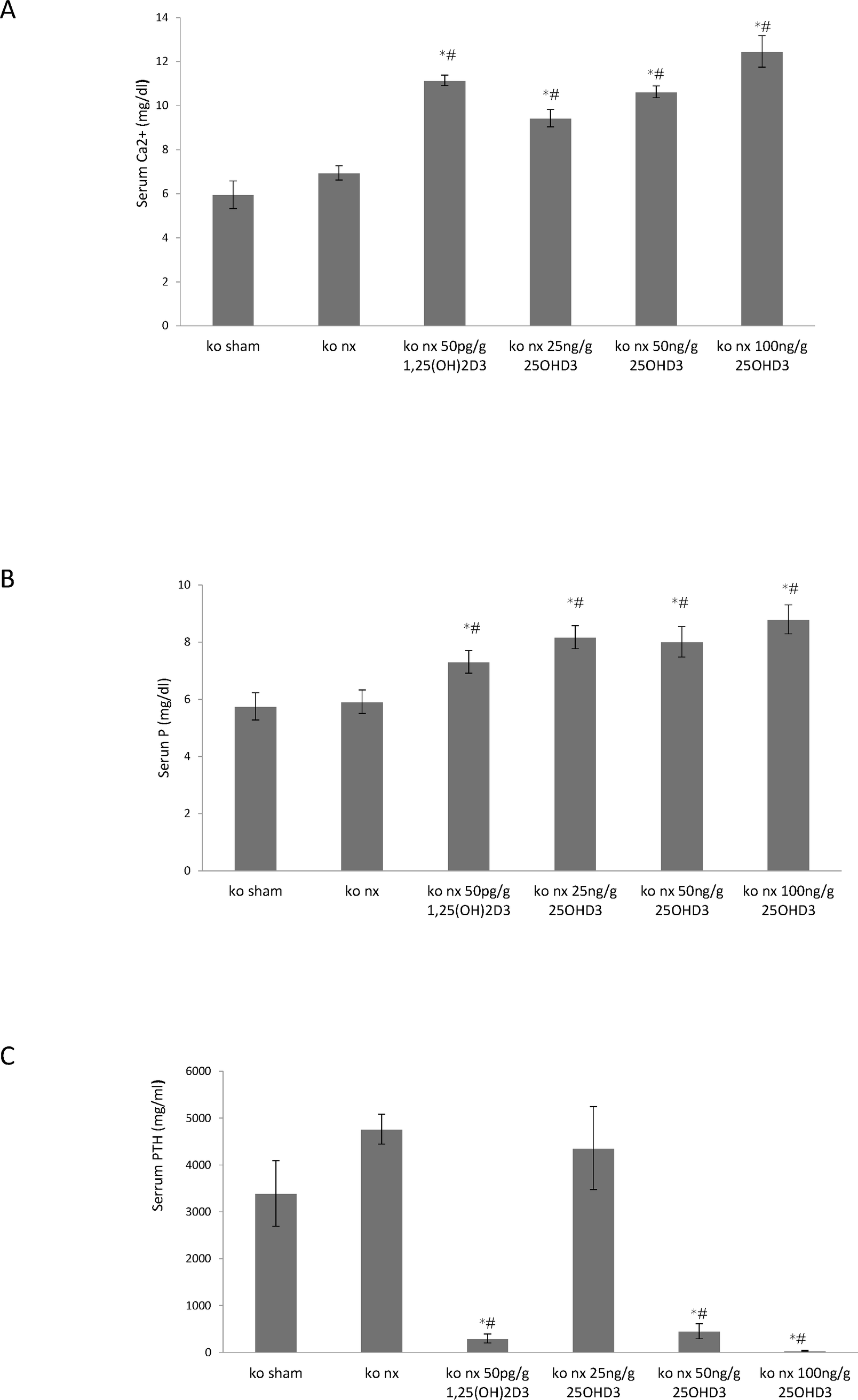

Supplement: S1 Fig — Levels of Ca (A), P (B) and PTH (C) in sham-operated KO animals, NX KO animals and NX KO animals treated with 50 pg/g of 1,25(OH)2D3 or 25, 50 and 100 ng/g of 25OHD3. *: p<0.01 vs KO sham. #: p<0.01 vs KO NX. (TIF) [file pone.0170654.s001.tif]
